# Enhanced Tau Protein Translation by Hyper-Excitation

**DOI:** 10.3389/fnagi.2019.00322

**Published:** 2019-11-20

**Authors:** Shunsuke Kobayashi, Toru Tanaka, Yoshiyuki Soeda, Akihiko Takashima

**Affiliations:** ^1^Department of Biochemistry, School of Pharmacy, Nihon University, Funabashi, Japan; ^2^Laboratory for Alzheimer’s Disease, Department of Life Science, Faculty of Science, Gakushuin University, Tokyo, Japan

**Keywords:** tau, glutamate stimulation, translation, synapse, phosphorylation

## Abstract

Tau is a microtubule-associated protein, localizing mainly in the axon of mature neurons. Phenotypic analysis of *Tau* knockout mice has revealed an impairment of synaptic plasticity but without gross changes in brain morphology. Since we previously described the presence of tau mRNA in the somatodendritic compartment, including the postsynapse, and demonstrated that it could be locally translated in response to glutamate, it appears that the regulated translation of synaptic tau can have a direct impact on synaptic function. Using SH-SY5Y cells, we herein confirm that glutamate dose-dependently regulates the translation of tau protein without altering tau mRNA levels. This is supported by the finding that cycloheximide blocks glutamate-stimulated increases in tau protein levels. Our observation that neural excitation can directly upregulate tau mRNA translation helps explain the pathological accumulation of tau in the somatodendrite.

## Introduction

Intracellular inclusions of hyperphosphorylated tau protein (neurofibrillary tangles, NFTs) and extracellular deposits of amyloid β (Aβ) are prominent neuropathological features in the brains of Alzheimer disease patients (Selkoe, 2004). The propagation of NFTs from the entorhinal cortex to the neocortex, followed by neuronal and synapse loss, corresponds closely with the temporal and clinical manifestation of AD symptoms – from impaired memory to dementia (Braak and Braak, 1991; Gomez-Isla et al., 1997; Iqbal and Grundke-Iqbal, 2002). Thus, the formation and propagation of NFT is likely to contribute to AD symptomatology.

In the healthy brain, tau is an exclusively axonal protein, engaged in the assembly and stability of microtubules (Weingarten et al., 1975). In contrast, in the AD brain, tau is hyperphosphorylated and forms fibrils that appear as neuropil threads in dendrites and as NFTs in the somato-dendritic compartment and axons (Kowall and Kosik, 1987). Evidence showing that NFT formation is preceded by a pre-tangle stage where non-fibrillar and hyperphosphorylated tau accumulates in the soma and dendrites of neurons (Gotz et al., 1995; Uchihara et al., 2001; Braak and Del Tredici, 2013) indicates that tau hyperphosphorylation occurs in the somatodendrite before its fibrillation and the appearance of neurofibrilar lesions.

Tau has recently been ascribed with a role in synaptic function in physiological conditions. For example, we showed that tau is essential for the induction of long-term depression (LTD) (Kimura et al., 2014), a phenomenon that can be explained by its presence in the somatodendritic compartment: non-axonal tau is translated from tau mRNA that is transported to the post-synapse as a complex comprised of mRNA binding protein (mRNP) and MyosinIV (Kobayashi et al., 2017). In line with previous work showing that neuronal excitation can trigger the local translation of other dendritic molecules implicated in synapse formation and plasticity (e.g., CaMKIIα, GluR, and Arc) (Steward and Halpain, 1999; Steward and Worley, 2002; Steward and Schuman, 2003; Bramham and Wells, 2007), we reported that glutamatergic stimulation enhances tau protein translation and the accumulation of hyperphosphorylated tau in somatodendrites of mouse hippocampal neurons (Kobayashi et al., 2017).

Our previous work (Kobayashi et al., 2017) was primarily based on immunohistochemical and immunoblotting analyses, approaches that allow visualization and quantitation of tau levels in neuronal dendrites, soma, and axons. The present experiments, performed on glutamate-stimulated human neuroblastoma SH-SY5Y cells, aimed at strengthening the evidence that accumulation of tau in somatodendritic compartment in disease states is due to enhancement of tau translation in response to glutamatergic stimulation.

## Results

**FIGURE 1:**
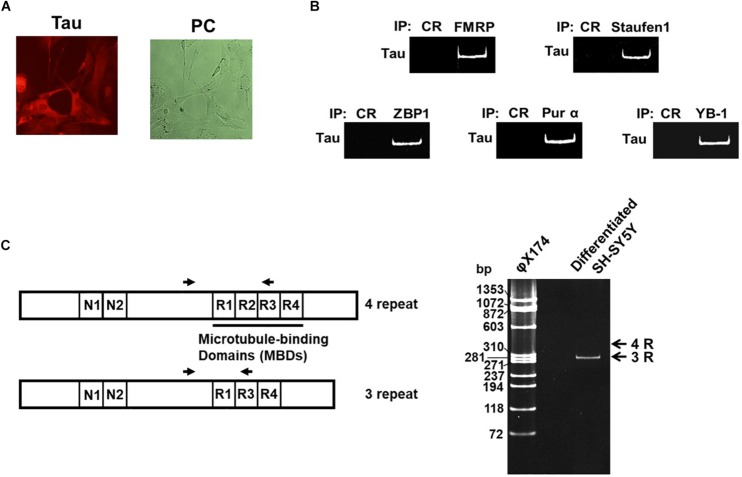
Tau mRNA interacts with mRNA-binding proteins and is expressed in SH-SY5Y cells. **(A)** SH-SY5Y cells were treated with RA for 4 days and immunostained with anti-tau antibody (Alexa Fluor 555) (left-hand image); the right-hand image shows a phase contrast (PC). **(B)** The cytosolic fraction of differentiated SH-SY5Y cells was immunoprecipitated with specific antibodies directed against dendritic mRNA-binding proteins, or control IgG. RNA was extracted from the immune complex, and double-stranded tau cDNA was detected by RT-PCR. **(C)** RNA was extracted from the cytosolic fraction and RT-PCR was performed using specific primers to examine the presence of the 3- or 4-repeat regions. The primer positions are shown as opposed arrows. φX174: molecular marker.

Cell bodies and neurites of neural SH-SY5Y cells differentiated with retinoic acid (RA) displayed tau immunoreactivity when stained with a pan-tau antibody (Figure 1A). To confirm the interaction of dendritic mRNA-binding proteins with tau mRNA, whole-cell extracts of differentiated SH-SY5Y cells were immunoprecipitated using specific antibodies against the dendritic mRNA-binding proteins FMRP (Greenough et al., 2001), Staufen (Kiebler et al., 1999), ZBP1 (Tiruchinapalli et al., 2003), Pur α (Ohashi et al., 2000), and YB-1 (Funakoshi et al., 2003; Tanaka et al., 2010). After extraction of RNA from the immunoprecipitants, RT-PCR using specific primers for a common region in six tau mRNA isoforms was performed (Figure 1B). That analysis revealed that Tau mRNA interacts with all of the dendritic mRNA-binding proteins of interest. We also examined which tau mRNA – 3-repeat tau or 4-repeat tau – is expressed in the differentiated cytosolic fraction of SH-SY5Y cells (Figure 1C) by RT-PCR assays, using specific primers to detect the region encoding the microtubule-binding domains (MBDs). Only one RT-PCR product with a length corresponding to 3-repeat tau was detected, supporting an earlier report (Uberti et al., 1997).

**FIGURE 2:**
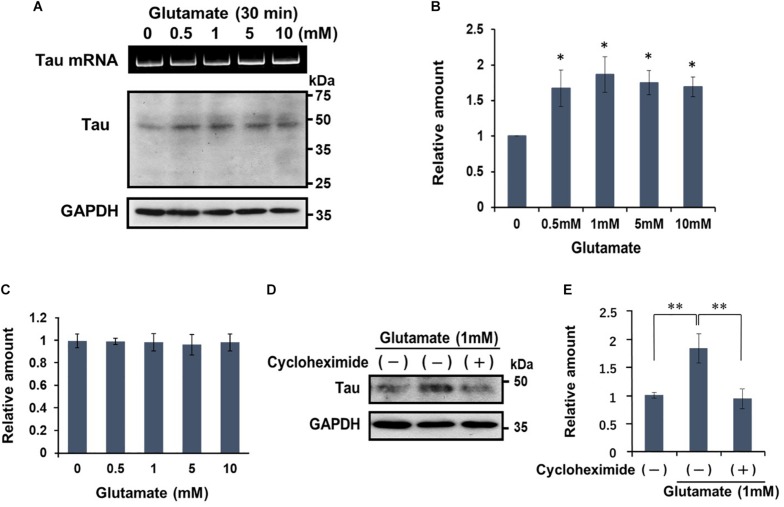
Glutamate stimulates the translation of tau mRNA. **(A)** Differentiated SH-SY5Y cells were treated with various concentrations of glutamate for 30 min, and cell extracts were examined for tau protein content by Western blotting. GAPDH served as a control. RT-PCR data of tau mRNA is also shown. **(B)** The signal intensity of tau protein was normalized to that of GAPDH, and expressed as a relative amount. Each value represents the mean and standard error obtained from four independent experiments. ^∗^*P* < 0.05 versus control (one-way ANOVA, followed by Tukey–Kramer *post hoc* test). **(C)** Quantitative RT-PCR of tau mRNA after exposure to various doses of glutamate concentration. **(D)** Differentiated SH-SY5Y cells were treated with glutamate (1 mM) for 30 min in the presence or absence of cycloheximide (20 μg/ml). Tau protein from each cell extract was analyzed by Western blotting. GAPDH was used as a loading control. **(E)** Levels of tau protein are expressed relative to that of GAPDH. Each value represents the mean and standard error obtained from four independent experiments. ^∗∗^*P* < 0.01 versus control or in the presence of CHX (one-way ANOVA, followed by Tukey–Kramer *post hoc* test).

Earlier work demonstrated that the excitotoxic effects of glutamate in SH-SY5Y only become manifest after extended (overnight) exposure to high doses of glutamate (Sun et al., 2010). This contrasts with the rapid (within 30 min) induction of translation of dendritic tau mRNA when hippocampal neurons are treated with 0.5 M of glutamate (Kobayashi et al., 2017). Here we show that neural activation with glutamate for 30 min dose-dependently upregulates tau protein levels, as measured by quantitative Western blotting (Figures 2A,B); tau protein levels plateaued at glutamate doses >1 mM (Figure 2B). Glutamate treatment did not alter tau mRNA expression (Figure 2C) and importantly, the glutamate-induced increase in tau protein levels was abolished when the cells were co-incubated with cycloheximide (Figures 2D,E); the latter indicates that glutamate activates the translation of tau mRNA.

**FIGURE 3:**
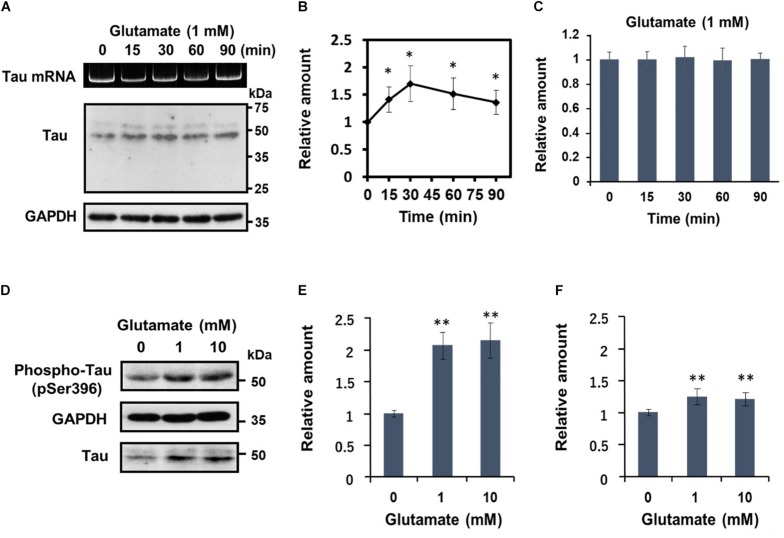
Glutamate-responsive translational activation of tau mRNA is transient, but induces AD-relevant phosphorylation of the newly synthesized tau protein. **(A)** Time course analysis of the glutamate-dependent increase of tau protein levels. Differentiated SH-SY5Y cells were treated with 1 mM glutamate for the time indicated and tau protein from each cell extract was analyzed by Western blotting. GAPDH served as a loading control. RT-PCR analysis of tau mRNA is also shown. **(B)** The signal intensity of tau protein at each indicated time point was expressed relative to that of GAPDH. Each value represents the mean and standard error obtained from five independent experiments. ^∗^*P* < 0.05 versus control (one-way ANOVA, followed by Tukey–Kramer *post hoc* test). **(C)** Quantitative RT-PCR of tau mRNA at each time point is shown. **(D)** Differentiated SH-SY5Y cells were treated with different concentrations of glutamate for 30 min, and total tau protein and phosphorylated tau (anti-tau pSer396) was detected by Western blotting. The amount of phosphorylated tau protein was expressed relative to that of GAPDH **(E)** or total tau **(F)**. Each value represents the mean and standard error obtained from four independent experiments. ^∗∗^*P* < 0.01 versus control (one-way ANOVA, followed by Tukey–Kramer *post hoc* test).

Subsequently, we undertook a detailed analysis of the time course of activity-dependent tau mRNA translation, using 1 mM glutamate (Figures 3A–C). Western blot analysis revealed that after peaking at 30 min after application of glutamate, tau protein levels declined gradually (Figure 3B). At no time point were the changes in tau protein accompanied by alterations in tau mRNA levels (Figure 3C). Another important observation from these experiments was that glutamate treatment resulted in a marked increase of phosphorylated tau bearing an AD-relevant epitope (detected by anti-tau pSer396 antibody) (Figures 3D,E). A significant upregulation of phosphorylated tau was also observable when results were normalized to total tau levels (Figure 3F), confirming that glutamate increases tau phosphorylation.

Together, these results demonstrate that glutamatergic stimulation leads to rapid *de novo* synthesis and phosphorylation of tau independently of tau mRNA transcription.

## Discussion

### Regulation of Tau Translation

In healthy, mature neurons, tau is predominantly localized in axons, with only low levels of expression in dendrites (Kanai and Hirokawa, 1995; Hirokawa et al., 1996; Ittner and Ittner, 2018). In contrast, somatodendritic levels of tau are significantly increased in AD and other tauopathies (Kowall and Kosik, 1987; Hoover et al., 2010). While the mechanisms responsible for the differential distribution of tau in healthy vs. diseased neurons are still unclear, one plausible mechanism for somatodendritic accumulation of tau is through the transport of tau mRNA in association with mRNP to the somatodendritic compartment where tau protein is synthesized *de novo* upon neuronal activation, e.g., by glutamate (Kobayashi et al., 2017).

Using differentiated human SHSY5Y cells, we here confirmed our previous observation in primary mouse hippocampal neurons (Kobayashi et al., 2017) that tau mRNA associates with dendritic mRNA-binding proteins, such as FMRP (Greenough et al., 2001), Staufen (Kiebler et al., 1999), ZBP1 (Tiruchinapalli et al., 2003), Pur α (Ohashi et al., 2000), and YB-1 (Funakoshi et al., 2003; Tanaka et al., 2010); as in hippocampal neurons, tau protein was visualized in both the cell body and neurites of SHSY5Y cells. Further, glutamate was found to stimulate tau mRNA translation into tau protein in a dose-dependent manner; maximum levels of tau protein were detectable at 30 min after application of glutamate after which they declined gradually (Figures 3A,B). Proteasomal or autophagic activity are likely to be responsible for the latter reductions in dendritic tau protein (see Balaji et al., 2018).

### Synaptic Tau

Although only transiently increased, it is highly plausible that the glutamate-stimulated translation of tau protein is of biological significance. Indeed, we previously reported that hippocampal LTD cannot be induced in mice lacking the *tau* gene (Kimura et al., 2014), suggesting a role for tau in synaptic plasticity and function. Furthermore, it has been reported that tau phosphorylation at Ser396 is required for LTD induction (Regan et al., 2015). Glutamate-induced phosphorylated tau in synapse may involve in synaptic plasticity.

While synaptic proteins, such as glutamate receptors and BDNF, are locally translated from their mRNA in the synaptic region (Kim et al., 2013; Leal et al., 2014), the origin of tau in synapses under physiological conditions has been somewhat controversial: although tau mRNA shares RNA binding proteins with synaptic protein mRNAs, and several authors reported the presence of presynaptic tau in human and animal brain tissue (Tai et al., 2012; Jadhav et al., 2015), other investigators failed to detect an overlap in immunolabeled endogenous mouse tau with dendritic proteins such as drebrin and microtubule-associated protein 2 (Kubo et al., 2019). Nonetheless, given the evidence that reducing tau levels has a major impact on synaptic function (Hoover et al., 2010; Kimura et al., 2014; Regan et al., 2015; Guo et al., 2017), a postsynaptic site of tau action seems highly likely. We suggest that synaptic tau may often elude detection because of its short lifespan.

### Implications of Synaptic Tau for Neurodegeneration

Neurofibrillary tangles are a well-known pathological marker for neurodegeneration in AD and the other tauopathies and, conversely, correlate with cognitive decline. However, NFTs themselves do not induce neurotoxicity; rather, tau aggregation processes seem to be responsible for synaptic degradation and cognitive dysfunction (Santacruz et al., 2005; Kimura et al., 2010; Takashima, 2013). Since the shift in localization of tau from a predominantly axonal site to somatodendritic site occurs during early stages of neurodegeneration (Zempel et al., 2010; Zempel and Mandelkow, 2014), the consensus now holds that tau aggregates start to form from accumulations of phosphorylated tau in the somatodendritic compartment. Although it is still unclear as to how somatodendritic phosphorylated tau accumulation contributes to neurotoxicity, several hypotheses appear to be tenable. For example, we previously reported that granular tau oligomers appear before tau fibril formation and that an inhibitor of tau aggregation blocks the formation of granular tau and prevents neuronal loss in a mouse model of tauopathy (Soeda et al., 2015). These findings suggest that aggregates of granular tau oligomers are responsible for inducing neurotoxicity. Moreover, tau dimerization is known to be a crucial initiating step in the neurodegenerative process in tau-expressing cells; this, in turn, further enhances tau aggregation and the production of reactive oxygen species (ROS) and cytoplasmic Ca^2+^ which ultimately trigger cell death (Pickhardt et al., 2017). While any cytoplasmic tau can precipitate such neurotoxicity, it is worthwhile noting, in the context of this study, that tau aggregates in the synapse can impair clathrin-mediated endocytosis (Hoover et al., 2010; Yu et al., 2019). The loss of synaptic function through the aggregation of tau can potentially inhibit the endocytosis and homeostatic balance of excitatory synaptic receptors, thus disrupting synaptic plasticity and ultimately triggering cell death.

## Materials and Methods

### Antibodies

Anti-tau (rabbit) antibody (catalog no. SC1996-R, lot no. B1213) was from Santa Cruz Biochemistry. Anti-tau pSer396 (rabbit) antibody (catalog no. BS4196, lot no. CJ36131) was from Bioworld Technology. Anti-FMRP (mouse) antibody (catalog no. MAB2160, lot no. 2137991, clone 1C3) was from Millipore. Rabbit anti-Staufen1 (catalog no. ab73478, Gr21579-1), mouse anti-Pur α (catalog no. ab77734, lot no. GR98153-2), and rabbit anti-YB-1 (catalog no. ab76149, GR221265-24, clone EP2708Y) antibodies were from Abcam. Anti-ZBP1/IMP1 (mouse) antibody (catalog no. RN001M, lot no. 001, clone 6H6) was from MBL Life Science. Alexa Fluor 555-conjugated goat anti-rabbit IgG (catalog no. A21428, lot no. 1937183) was purchased from Thermo Fisher Scientific. Horseradish peroxidase (HRP)-linked anti-rabbit IgG (donkey) (catalog no. NA934V. lot no. 377022) was from GE Healthcare Life Science.

### Cell Culture and Immunocytochemistry

Human neuroblastoma SH-SY5Y cells were grown in Dulbecco’s modified Eagle medium with 10% fetal bovine serum. Cells were differentiated by incubation with 33.3 μM RA for 4 days. For immunocytochemistry, the cells were fixed in 4% paraformaldehyde in phosphate-buffered saline (PBS) for 10 min, treated with 0.5% Triton X-100 in PBS for 15 min, and then incubated with anti-tau antibody in PBS containing 5% skimmed milk at room temperature for 2 h. After washing with PBS, specimens were incubated with Alexa Fluor 555-conjugated second antibody for 1 h, washed with PBS, and viewed with an Olympus inverted microscope linked to a DP-70 imaging system.

### Western Blot Analysis

Cells were lysed in TKM buffer containing 50 mM triethanolamine (pH 7.8), 50 mM MgCl_2_, 0.25 M sucrose, 1 mM phenylmethylsulfonyl fluoride (PMSF), 1 mM dithiothreitol (DTT), protease inhibitors (complete cocktail, Roche), 1× phosphatase inhibitor cocktail solution (Wako Pure Chemical Industries), and ribonuclease inhibitor (0.2 unit/μl, Takara Bio Inc.). The lysate was centrifuged at 3,000 rpm for 10 min and the supernatant was used as the cytosol fraction. Proteins were separated by SDS-PAGE and transferred to a Polyvinylidene difluoride (PVDF) membrane. After treatment with anti-tau antibody, the membrane was incubated with a secondary HRP-conjugated antibody. Protein signals were detected with an ECL kit (GE Healthcare Life Science) and assessed by densitometric analysis.

### Immunoprecipitation and RT-PCR

Each antibody (2 μg) was bound to Dynabeads Protein G (Life Technologies) and incubated with cell lysate at 4°C for 4 h. The beads were washed with PBS containing 0.1% BSA and co-immunoprecipitated RNAs were extracted with SDS–phenol–chloroform and dissolved in water. First-strand cDNA was synthesized with Moloney Murine Leukemia Virus (MMLV) reverse transcriptase (Takara Bio Inc.) using an oligo (dT) primer. Double-stranded tau cDNA was synthesized using specific primers. The RT-PCR products were stained with ethidium bromide and analyzed using a gel documentation system (BioRad GelDoc XR Plus ImageLab). Primer pairs were: 5′-ACTGGCATCTCTGGAGTGTGTG-3′ (forward) and 5′-GCAGCTACAAGCTAGGGTGCAAG-3′ (reverse). To investigate which tau mRNA – 3-repeat tau or 4-repeat tau – is expressed in differentiated SH-SY5Y cells, RT-PCR was performed using specific primers with the following sequences: 5′-AGGTGAACCTCCAAAATCAGGGGATC-3′ (forward) and 5′-ACADTTGGAGGTCACTTTGCTC-3′ (reverse).

### Quantitative RT-PCR (Real-Time qRT-PCR)

Total RNA was extracted with a mini-prep RNA extraction kit (QIAGEN) in accordance with the manufacturer’s instructions, and first-strand cDNA was synthesized from 1.0 μg of total RNA using reverse transcriptase (Takara Bio Inc.) as described above. Aliquots of cDNA were used for qPCR with a StepOnePlus Real Time PCR system (Applied Biosystems) using PowerUp SYBR Green Master Mix (Thermo Fisher Scientific). The level of tau mRNA expression was normalized to that of β-actin mRNA. The primer sequences used for double-stranded tau cDNA are given above. The primers for glyceraldehyde-3-phosphate dehydrogenase (GAPDH) mRNA were as follows: 5′-TGAGTACGTCGTGGAGTCCACTG-3′ (forward) and 5′-GGGATGATGTTCTGGAGAGC-3′ (reverse).

## Data Availability Statement

The raw data supporting the conclusions of this manuscript will be made available by the authors, without undue reservation, to any qualified researcher.

## Author Contributions

AT and SK made conception and design of this work. TT and YS collected and analyzed the data. SK drafted the manuscript. AT critically revised and finalized the manuscript.

## Conflict of Interest

The authors declare that the research was conducted in the absence of any commercial or financial relationships that could be construed as a potential conflict of interest.
